# The 2010 structural-demographic forecast for the 2010–2020 decade: A retrospective assessment

**DOI:** 10.1371/journal.pone.0237458

**Published:** 2020-08-17

**Authors:** Peter Turchin, Andrey Korotayev

**Affiliations:** 1 Complexity Science Hub Vienna, Vienna, Austria; 2 University of Connecticut, Storrs, CT, United States of America; 3 National Research University Higher School of Economics, Moscow, Russia; 4 Institute of Oriental Studies, Russian Academy of Sciences, Moscow, Russia; University Of Bristol, UNITED KINGDOM

## Abstract

This article revisits the prediction, made in 2010, that the 2010–2020 decade would likely be a period of growing instability in the United States and Western Europe Turchin P. 2018. This prediction was based on a computational model that quantified in the USA such structural-demographic forces for instability as popular immiseration, intraelite competition, and state weakness prior to 2010. Using these trends as inputs, the model calculated and projected forward in time the Political Stress Indicator, which in the past was strongly correlated with socio-political instability. Ortmans et al. Turchin P. 2010 conducted a similar structural-demographic study for the United Kingdom. Here we use the Cross-National Time-Series Data Archive for the US, UK, and several major Western European countries to assess these structural-demographic predictions. We find that such measures of socio-political instability as anti-government demonstrations and riots increased dramatically during the 2010–2020 decade in all of these countries.

## Introduction

How resilient are our societies to internal and external shocks? Can we model and forecast the dynamics of social resilience and its opposite, social breakdown? A major research challenge in answering this question is that growing socio-political instability results from multiple interacting factors: economic, political, and cultural. Previous work on this important issue has been conducted largely by political theorists, policy analysts, sociologists, historians, and computational modelers who worked in isolation from each other with focused, domain-specific data sources [for a recent review, see 1]. Separately, they all offer intriguing insights and have produced important discoveries, but ultimately each can provide only one piece of the puzzle. Structural-demographic theory (SDT) offers a more wholistic framework for investigating such multiple interacting forces that shape long-term social pressures that lead to revolutions, civil wars, and other major outbreaks of socio-political instability. Furthermore, SDT can be, and has been formulated as an explicit computational model capable of forecasting future quantitative dynamics of social unrest and political violence in specific social systems. In this article we provide an assessment for a SDT forecast made ten years ago about the United States and Western Europe [2].

Structural-demographic theory (SDT) was proposed by Jack Goldstone [3, 4] and further developed and tested by an international crew of investigators, including Nefedov [5, 6], Turchin [7–10], Turchin and Nefedov [11], Korotayev et al. [12, 13]. The SDT proposes that the causes of revolutions and major rebellions are in many ways similar to processes that cause earthquakes [3]. In both revolutions and earthquakes it is useful to distinguish “pressures” (structural conditions, which build up slowly) from “triggers” (sudden releasing events, which immediately precede a social or geological eruption).

**Fig 1:**
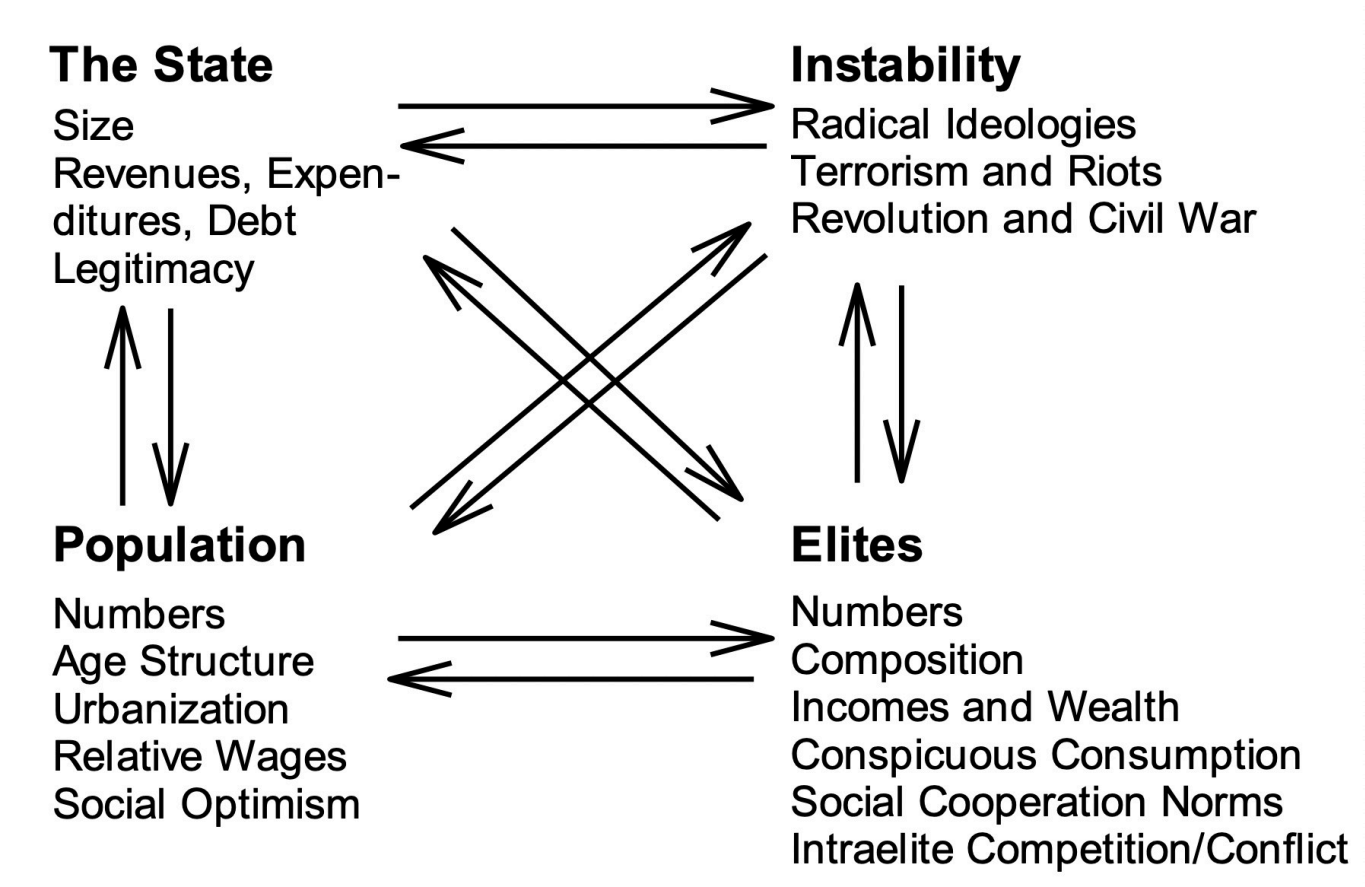
The main logical components of the structural-demographic theory [9].

Specific triggers of political upheavals are difficult, perhaps even impossible to predict. On the other hand, structural pressures build up slowly and more predictably, and are amenable to analysis and forecasting. Furthermore, many triggering events themselves are ultimately caused by pent-up social pressures that seek an outlet—in other words, by the structural factors. The main focus of SDT (as the name implies) is on the structural pressures undermining social resilience. The theory represents complex human societies as systems with three main compartments (the general population, the elites, and the state) interacting with each other and with socio-political instability via a web of nonlinear feedbacks (Fig 1). The focus on only these four structural components is not quite as great oversimplification as it may appear, because each component has a number of attributes that change dynamically in response to changes in other structural-demographic variables [see 9, 10].

In 2010 one of us (PT) used the SDT to make the following forecast: “The next decade is likely to be a period of growing instability in the United States and western Europe” [2]. This forecast was not simply a projection of the current trend in social instability into the future. As we shall see below (*Results*), social instability in major Western countries had been, in fact, declining prior to 2010. Rather, the basis for this forecast was a quantitative model that took as inputs the major SD drivers for instability (immiseration, intraelite competition, and state (in)capacity) and translated them into the Political Stress Indicator (PSI), which is strongly correlated with socio-political instability [3, 9]. The rising curve of the calculated PSI, then, suggests a growing future socio-political instability.

SDT is a general theory that guides our understanding of political violence dynamics and social breakdown in all large-scale state-level societies. However, there are sufficient institutional and other differences between different states. Thus, when we aim to analyse and, possibly, forecast instability dynamics in any particular state, we need to translate the general theory into a specific computational model tailored to the focal state. Over the past four decades this has been accomplished for a large, and growing, number of historical case-studies, ranging from Ancient empires to Early Modern states and nineteenth century’s revolutions and civil wars [3, 5–8, 10–14]. In addition to such historical tests, the theory has been applied to two contemporary societies. The first one is a structural-demographic model for the contemporary USA, which provided the basis for the 2010 prediction. The details of the USA structural-demographic model were published in Turchin [9] and later expanded into a book-length treatment [10]. The second study examined structural-demographic pressures for instability in the contemporary UK [15]. Both studies forecast growing social and political instability in the US and UK into the 2020s. In the next section (*Methods*) we first describe the SD model for forecasting social pressures for instability and next proceed to examining the empirically observed trends in socio-political instability so that we can assess how these predictions fared.

## Materials and methods

### Forecasting model

The model that we used for forecasting social pressures for instability was proposed by Jack Goldstone, who used it in his investigation of revolutions and rebellions in the early modern world [3]. His results showed that the PSI serves as a leading indicator of an outbreak of major political violence in his most detailed historical case study—the English Civil War. Goldstone also showed that the method works for the French Revolution of 1789 and the nineteenth-century revolutions in France and Germany. The model was further elaborated by Turchin in a series of publications [7, 9, 10]. In particular, he used the model to quantify social pressures toward instability in the period preceding the American Civil War and in the contemporary America. Here we summarize the specific version of the model that was used to forecast the dynamics of contemporary USA [9]. This is also the version that Ortmans et al. [15] used for the contemporary UK.

The key output variable in the model is the Political Stress Indicator (PSI or *Ψ*), which summarizes the structural-demographic pressures for instability (or, equivalently, the loss of social resilience of the modeled state). The PSI is modeled as a product of three entities (corresponding to the *Population*, *Elites*, and *the State* structural-demographic compartments, see Fig 1):

Ψ=MMP×EMP×SFD

Here MMP, or Mass Mobilization Potential, captures the effect of growing popular immiseration, EMP, or Elite Mobilization Potential, quantifies intra-elite competition and conflict, and SFD, or State Fiscal Distress, measures the weakening of the state. The first PSI component is:

$$\mathrm{MMP}=w^{-1}\frac{N_{\mathrm{urb}}}{N}A_{20-29}$$

where *w* is relative wage (the wage scaled by GDP per capita). Thus, *w*^–1^ is the inverse relative wage (a measure of economic distress). The urbanization index *N*_urb_/*N* is the proportion of total population (*N*) within the cities (*N*_urb_). The last term, *A*_20–29_, is the proportion of the cohort aged between 20 and 29 years in the total population, which reflects the role of “youth bulges” in the genesis of instability waves.

The second component of *Ψ* deals with the elite overproduction and competition:

$$\mathrm{EMP}=\varepsilon^{-1}\frac{E}{sN}=\frac{1}{s}\varepsilon^{-1}e$$

The first term on the right-hand side, *ε*^–1^, is the inverse relative elite income (average elite income scaled by GDP per capita), which is analogous to *w*^–1^ of the working population. High *ε*^–1^ (and low *ε*) can result either from too small a pie that the elites divide among themselves, or too many elites dividing the pie, leading to a high level of intraelite competition. Thus, *ε*^-1^ is a measure of intraelite competition in the economic domain. The second term measures the effect of intraelite competition in the political domain, specifically for government offices. It assumes that the demand for elite positions is proportional to the elite numbers, *E*. The supply of such positions will grow in proportion to the total population (*N*). The proportionality constant *s* is the number of government employees per total population (which is allowed to change dynamically). We further define relative elite numbers (relative to the total population) as *e = E/N*. Assuming that *s* doesn’t change too much, the dynamics of EMP will be primarily driven by the product, *ε*^-1^*e*, which reflects two aspects (economic and political) of elite overproduction and intraelite competition.

The dynamics of the relative elite numbers, *e*, is governed by the following differential equation:

$$\frac{de}{dt}=\mu_{0}\frac{w_{0}-w}{w}$$

where *w* is again the (worker) relative wage and *μ*_0_ and *w*_0_ are scaling parameters. Parameter *μ*_0_ modulates the magnitude of response in social mobility to the availability of surplus (*w*_0_ –*w*). Parameter *w*_0_ is the level at which there is no net upward mobility (when *w* = *w*_0_, *de/dt* = 0). In other words, the rate of change of relative elite numbers is the net rate of social mobility [9].

Relative elite income, *ε*, is calculated by assuming that the elites divide among themselves the amount of surplus produced by the economy, that is, GDP minus the share going to workers. This is divided by the number of elites, *E*, and scaled by GDP per capita. As is shown in Ref. [9], the expression for *ε* simplifies to:

$$\varepsilon =\frac{1-w\lambda }{e}$$

where *w* is the relative wage, *e* is the elite numbers relative to the population, and *λ* is the proportion of the population in the labor force.

The third component of *Ψ*, State Fiscal Distress, has two parts:

$$\mathrm{SFD}=\frac{Y}{G}(1-T)$$

The first one is national debt (*Y*) scaled in relation to the GDP (*G*). The second part measures the degree of (dis)trust that the population and elites have in the state institutions (including its ability to service the debt). This variable is related to a more general variable, the state legitimacy. Thus, *T* is the proportion of the population expressing trust, and (1 –*T*) is the proportion expressing *distrust* in the state institutions.

Most of the quantities in this equation can be estimated directly. Thus, the main component of MMP, the relative wage *w*, is the worker wage scaled by GDP per capita [data source: 16]. As for the other components of MMP: the urbanization rate, *N*_urb_*/N*, is given in the *Historical Statistics of the United States* [17] and the youth bulge index, *A*_20–29_, was obtained from the US Census Bureau. The EMP components (relative elite numbers, *e*, and relative elite incomes, *ε*) were calculated using the formulae given above, with parameters *μ*_0_, *w*_0_, and *λ* estimated from the data [9: fig 11]. National debt scaled by GDP data are from the US Department of the Treasury. Distrust in government data are taken from the Pew Research Center (proportion responding ‘some of the time’ or ‘never’ to the question, “How much of the time do you trust the government in Washington?”). The R scripts performing calculations and data are provided as supplementary online materials.

**Fig 2:**
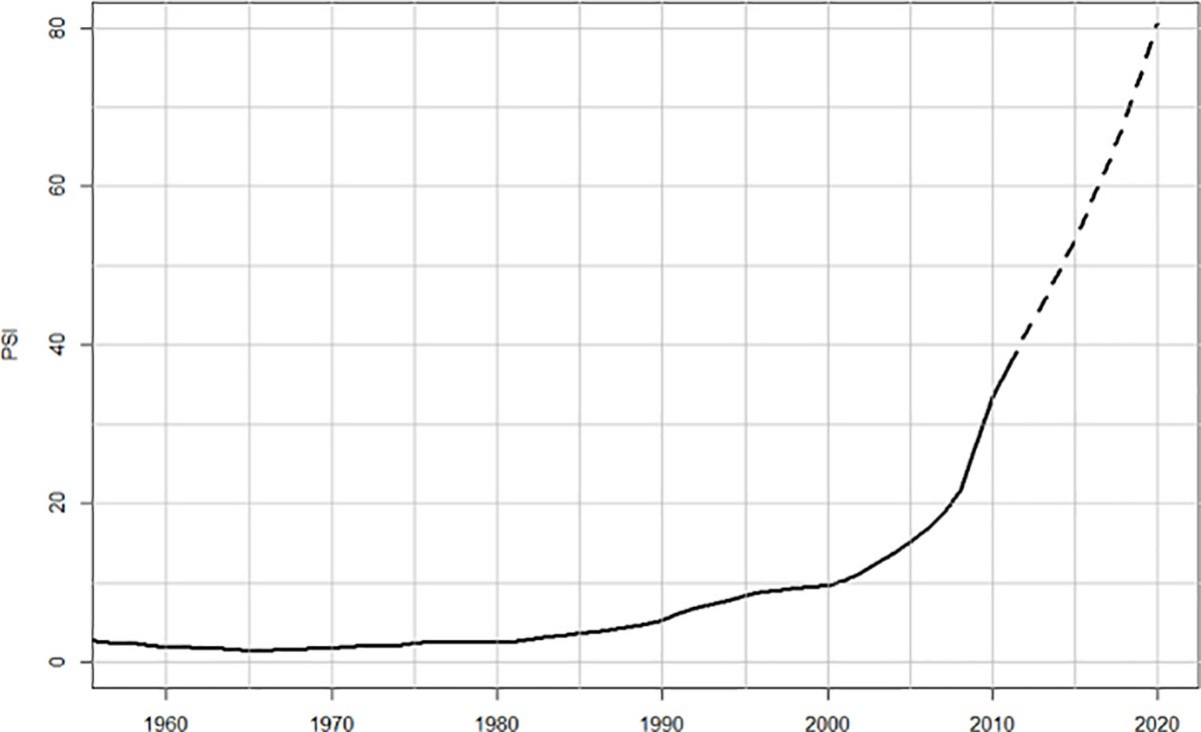
Calculated PSI for 1958–2011 (solid curve) and forecasted PSI for 2012–2020 (broken curve).

Fig 2 plots the dynamics of the PSI from 1958 (this starting point is due to data on trust in government institutions (*T*) being available only from 1958, when the Pew Research Center conducted its first study of this key social indicator). Let us restate what the PSI tells us, as well as its limitations as the predictor of socio-political instability. The PSI quantifies social pressures for instability arising from the three main structural-demographic processes, popular immiseration, intra-elite conflict, and fragility of the state. It is important to keep in mind that, as we acknowledged in the Introduction, socio-political instability results from multiple interacting factors. The PSI, for example, doesn’t take into account external influences on the focal state: geopolitical (for example, powerful neighbors fomenting insurrection), geoeconomic (for example, a spike in food prices in the world markets), or geocultural (a successful revolution in a culturally similar country). Furthermore, the PSI focuses on *secular waves* [11], long-term oscillations with periods of around 2–3 centuries. Yet, our historical research has shown that there is an additional process (which is not part of the Structural-Demographic Theory) that needs to be taken into account when studying sociopolitical instability: a shorter-term oscillation with an approximate period of 50 years, which has been dubbed the “fathers-and-sons” cycles [7, 18]. The dynamical interaction between longer structural-demographic, secular cycles, and shorter 50-year cycles was modeled and statistically analyzed in Chapter 2 of *Ages of Discord* [10]. Here we only wish to point out these additional complications, which we chose not to model explicitly. Our reasons are three-fold.

First, there is a value in a more parsimonious model, which focuses only on the structural-demographic forces. The structural-demographic drivers are more fundamental in the prediction of the dynamics of social resilience and its opposite, fragility. They operate in all known state-level societies, unlike the fathers-and-sons cycles (for example, Chinese data lack the 50-year cyclic component). Additionally, favorable structural-demographic conditions can suppress the fathers-and-sons spikes of violence, as apparently happened for the (missing) 1820 peak in the US [10]. Second, the 2010 forecast [2] was based on this parsimonious model, and we should stick with it to avoid contaminating the prediction with later developments. Third, the 50-year cycle, which spiked in the United States in c.1870, c.1920, and c.1970 was due to spike again around 2020. In other words, the father-and-sons cycle is expected to exacerbate the social pressures for instability, rather than change the forecast qualitatively. For further discussion of the interaction between these two drivers of instability we refer the reader to Ref. [10].

### Data analysis

We use the Cross-National Time-Series Data Archive [19] as our source of empirical data. The Cross-National Time Series (CNTS) database is a result of data compilation and systematization started by Arthur Banks in 1968 at the State University of New York Binghamton. The work was based on generalizing the archive of data from *The Statesman's Yearbooks*, published since 1864. It contains approximately 200 indicators for more than 200 countries. The database contains yearly values of indicators starting from 1815 excluding the periods of World Wars I and II (1914–1918 and 1939–1945).

CNTS database is structured by sections, such as territory and population, technology, economic and electoral data, internal conflicts, energy use, industry, military expenditures, international trade, urbanization, education, employment, legislative activity, etc.

In our paper we take a close look at the data describing internal conflicts (*domestic*). This section includes data starting from 1919 based on the analysis of events in eight various subcategories, which are used to compile integral Index of Sociopolitical Destabilization. We focus on two variables: riots (*domestic6*) and anti-government demonstrations (*domestic8*). An *anti-government demonstration* is defined as any peaceful public gathering of at least 100 people for the primary purpose of displaying or voicing their opposition to government policies or authority, excluding demonstrations of a distinctly anti-foreign nature. A *riot* is any violent demonstration or clash of more than 100 citizens involving the use of physical force. We focus on these two variables because other indices of instability in CNTS, such as *major government crises*, *purges*, and *revolutions* are rare or not present in mature Western democracies, and thus do not provide enough data for robust statistical characterization of temporal trends.

As the compilers of the CNTS database acknowledge, their data are subject to a variety of biases. Thus, we first check the CNTS data against an independent data set, derived from the US Political Violence Database [8]. Note that we don’t expect close agreement between these two databases because (1) they use different definitions of an instability event to be included and (2) while their sources partially overlap (both use the New York Times archives), the USPVD also includes a much broader variety of additional sources [see 8: Methods for details].

**Fig 3:**
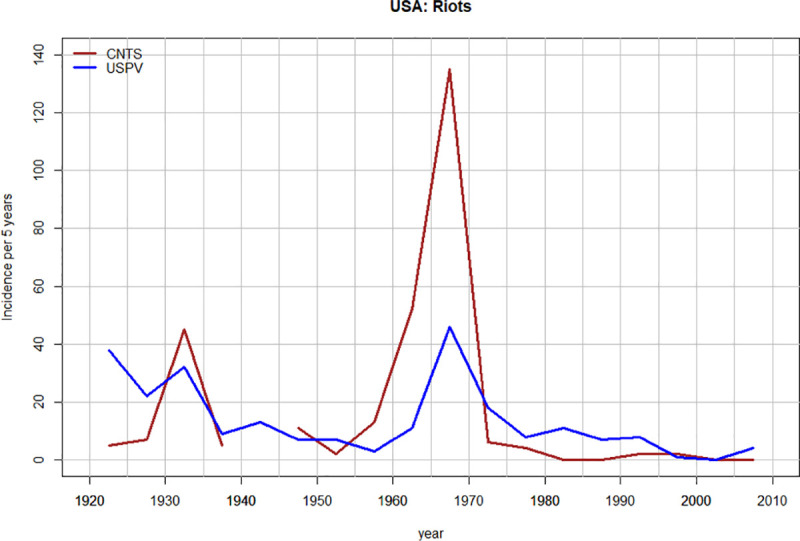
The number of riots per 5-year interval in the USA between 1920 and 2010: A comparison between CNTS data (dark red) and USPV data (blue).

A comparison between these two independently compiled databases shows that, while disagreeing in detail, they capture similar broad trends in US socio-political instability between 1920 and 2010 (Fig 3). In particular, both capture the instability peak of the late 1960s, which is followed by a decline to 2010.

## Results

### USA

**Fig 4:**
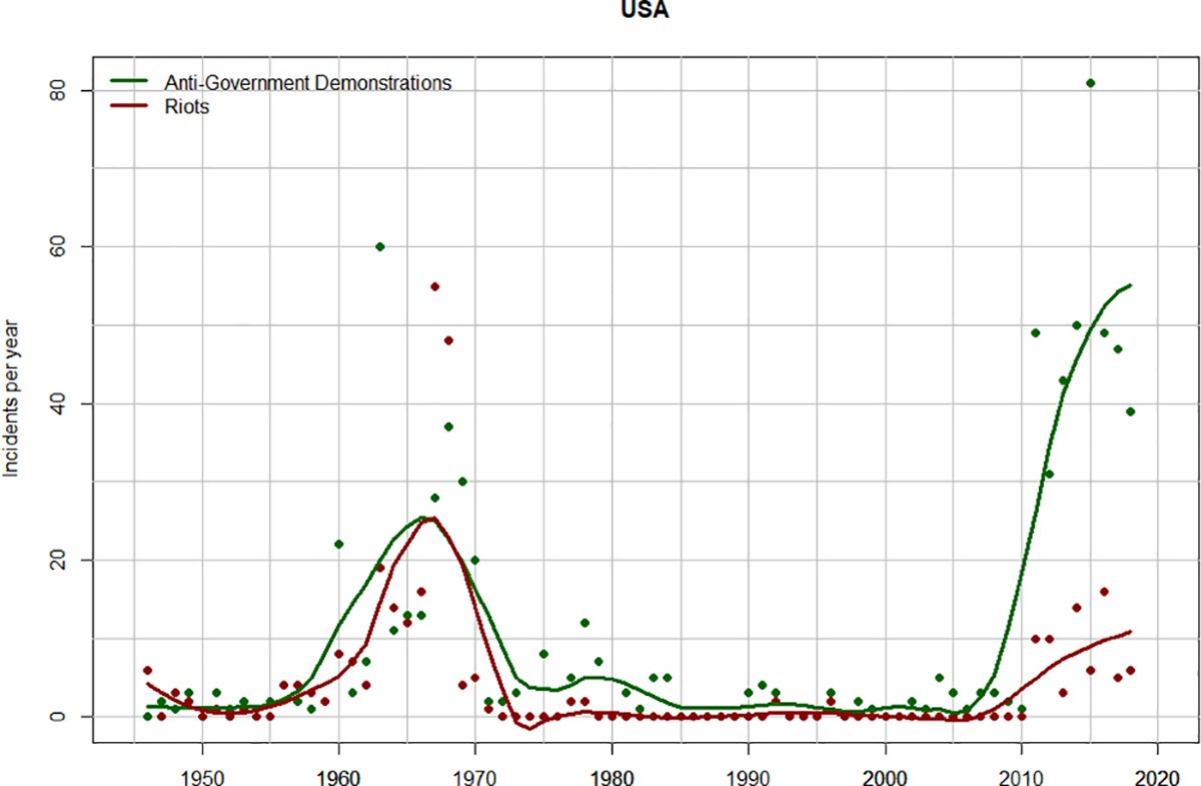
Temporal trends in anti-government demonstrations (green) and riots (dark red) in the USA, 1946–2018. The points show the number of incidents (demonstrations or riots) per year. The curves are data smoothed with the R function “loess” (span = 0.2).

Both anti-government demonstrations and riots exhibit similar temporal dynamics: a spike during the late 1960s followed by a low-instability regime until 2010, and then another spike after 2010 (Fig 4). However, while violent riots tend to dominate the 1960s spike, peaceful demonstrations are five times more frequent than violent riots after 2010.

### UK

**Fig 5:**
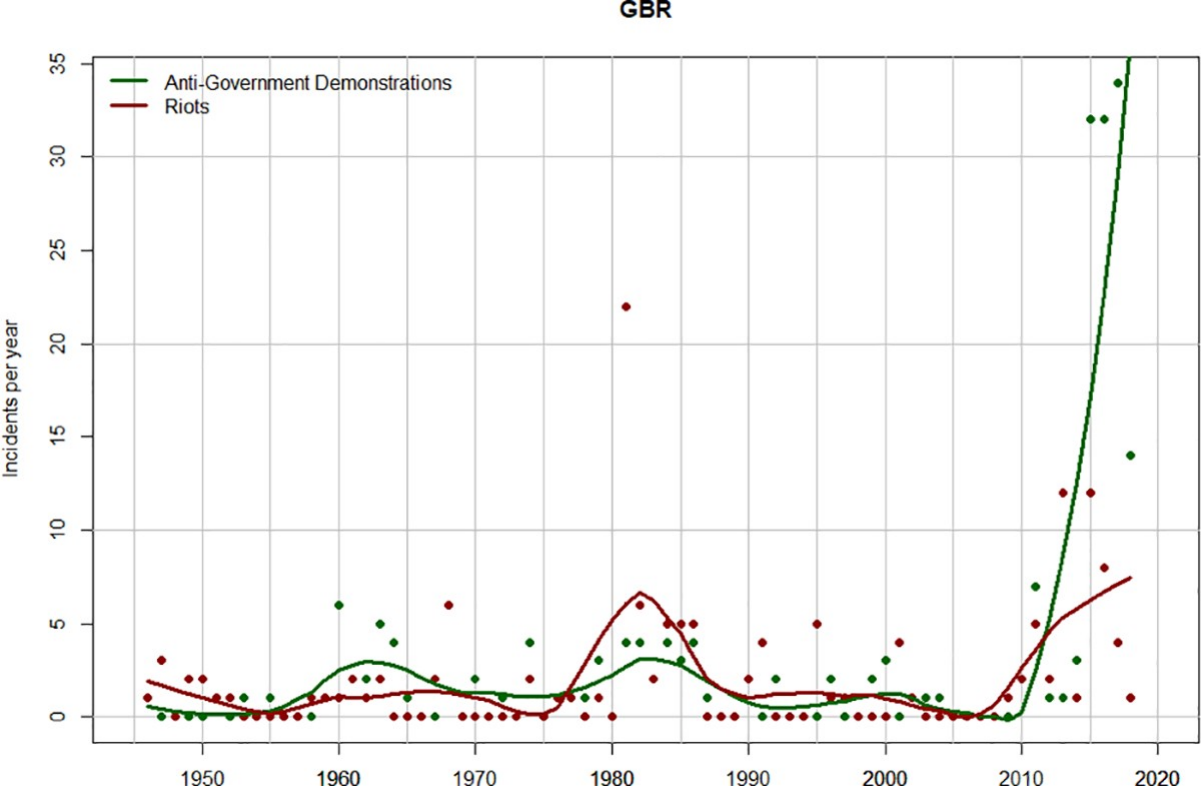
Temporal trends in anti-government demonstrations (green) and riots (dark red) in the UK, 1946–2018. The points show the number of incidents (demonstrations or riots) per year. The curves are data smoothed with the R function “loess” (span = 0.2).

In the United Kingdom the pre-2010 dynamics differ from those in the USA in that the previous peak of instability occurs later (during the early 1980s) and is not as prominent as the late 1960s peak in the USA (Fig 5). However, the rise in both riots and demonstrations after 2010 mirrors closely the USA pattern. Additionally, we see the same shift from more violent to less violent forms of protest. During the early 1980s riots were about two times as frequent as demonstrations, while during the 2010s demonstrations were much more frequent than riots.

## Discussion

### The 2010 prediction was accurate

Our retrospective assessment of the structural-demographic prediction for 2010–2020 shows that socio-political instability in the United States, indeed, increased sharply during this decade. The incidence per year of both non-violent (anti-government demonstrations) and violent (riots) events increased by an order of magnitude after 2010. The dynamics of these two indicators in the United Kingdom followed the same pattern: a decline to very low numbers before 2010 followed by a sharp spike after 2010. Thus, we conclude that the structural-demographic models accurately predicted future dynamics in these two countries.

**Fig 6:**
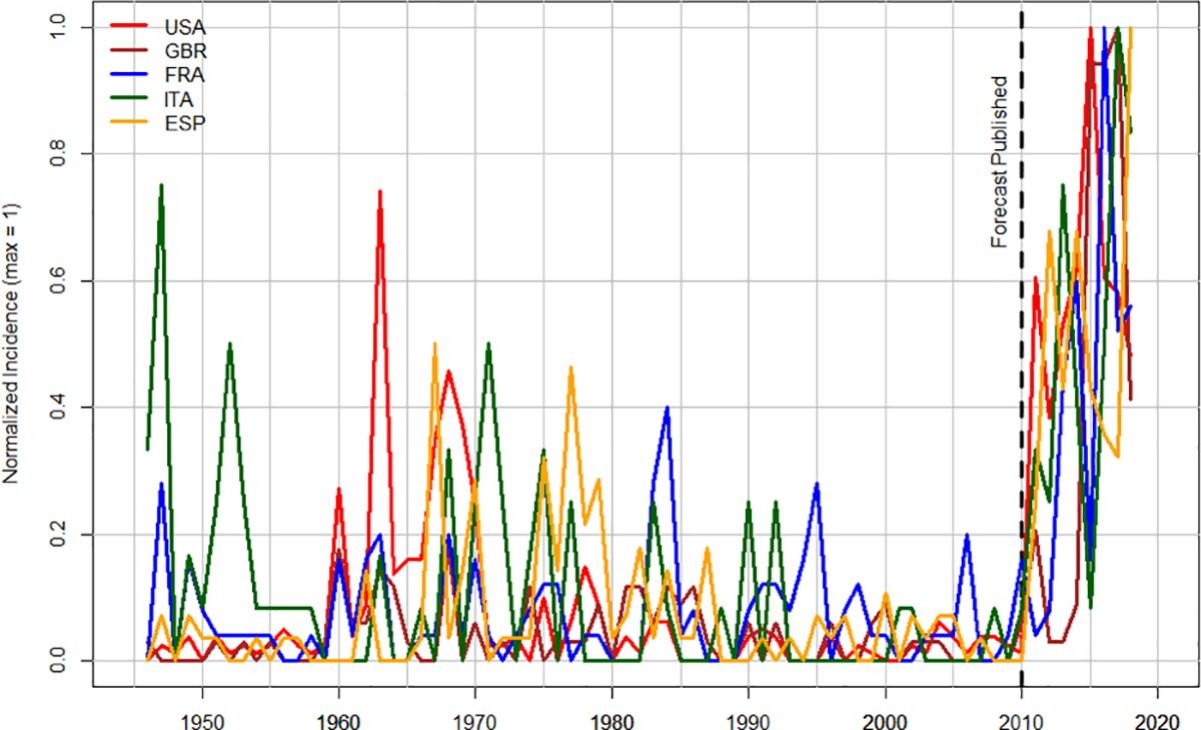
Temporal trends in anti-government demonstrations in five Western countries, 1946–2018. “Normalized Incidence” is the incidence of anti-government demonstrations per year scaled so that maximum for each country = 1. The vertical broken line indicates the year when the forecast was made (2010).

**Fig 7:**
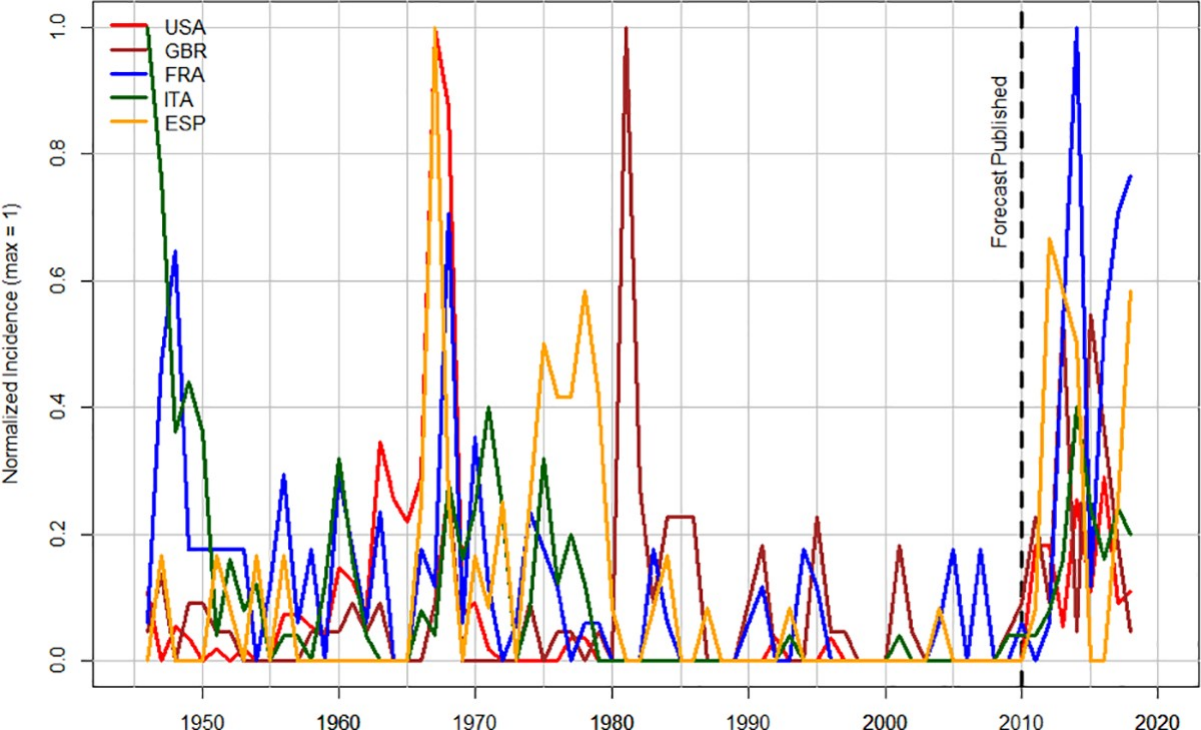
Temporal trends in riots in five Western countries, 1946–2018. “Normalized Incidence” is the incidence of riots per year scaled so that maximum for each country = 1. The vertical broken line indicates the year when the forecast was made (2010).

While we currently have fully developed quantitative SD models only for USA and UK, it is clear that the post-2010 increases in socio-political instability also affected a number of other Western countries. Figs 6 and 7 compare the trends in US and UK to three other major Western countries: France, Italy, and Spain (we omit Germany because its unification in 1990 introduces additional complications into the analysis). It is interesting that while pre-2010 peaks of instability vary substantially between different countries, the dynamics after 2010 are remarkably similar.

Anybody who follows world news couldn’t miss the dramatic increase in socio-political instability during the past decade. But in 2010 the perception of where the world was moving was the opposite. As Fig 6 shows, by 2010 violent expressions of instability had been on decline for 25–40 years, depending on the country. Seemingly nothing presaged the outbreak of violence that we saw after 2010. As a result, a number of pundits and public intellectuals declared an end of violence, at least in the mature Western democracies. The most outspoken was Steven Pinker, who in *The Better Angels of Our Nature*: *Why Violence Has Declined*, researched and written during the “noughties” (2000s) and published in 2011, argued that violence in the world has declined both in the long run and in the short run. The past decade has demonstrated that this assessment was wrong, when we look at a decadal time scale and a particular kind of violence—collective violence within states (see Fig 7 above, as well as, e.g., [15, 20–22]). This focus on intrastate collective violence (while leaving personal violence, e.g. homicide, and interstate warfare for other analyses) is determined by the main variable that the structural demographic theory focuses on explaining [see 8 for discussion].

To structural-demographic analysts the trend reversal in socio-political instability was not a surprise for two reasons. First, historical analysis shows that peaceful periods lasting one or two generations (25–50 years) are very common in history. They tend to occur during the “integrative phases” of structural-demographic cycles [11]. They are invariably (at least in all historical cases that have been studied in detail so far) succeeded by disintegrative phases, characterized by surging collective violence, state breakdown, and recurrent civil war.

Second, our understanding of why instability trends are periodically reversed, resulting in an alternation of integrative and disintegrative phases, is much more sophisticated than simply an appeal to “cyclic history.” In fact, the oscillations between relatively peaceful and violent periods are not cycles with fixed periods. Rather, these somewhat irregular oscillations arise as a result of dynamical feedbacks that affect the functioning of social systems. In other words, if we want a reliable forecasting tool for when the next outbreak of violence occurs, what we *don’t want to do* is count how many years have passed since the last such outbreak. Instead, we want to quantify the structural pressures for instability. The SDT provides us with such a tool, and the 2010 forecast used this insight from the SDT. The success of this forecast strengthens the empirical support for the SDT. It also suggests that the SDT is not simply a theory about historical societies; structural-demographic mechanisms continue to operate today even in mature Western democracies. However, the specific models we develop for contemporary states must take into account the many ways in which they differ from historical pre-industrial states and empires. And, as was pointed out earlier, SDT models need to be tailored to each specific country, due to the variation in their institutional makeup.

### The significance of our results for the broader structural-demographic studies

Structural-demographic theory was proposed 30 years ago [3]. Although it was successively refined by other theorists, including the authors of this article [9–11, 13, 23–26], the theoretical core, and especially the emphasis on intra-elite competition and conflict as the most important driver of socio-political instability and state breakdown, remained constant. Over the past three decades, the theory was empirically tested by a growing number of researchers. Currently, there are detailed investigations of at least twenty crises, in which researchers brought together multiple quantitative data sets to test the predictions of the theory (see Table 1). Additionally, there are several dozen other less detailed examples. The overall verdict is that theory’s predictions are well supported by data. At the same time, rival theories are not supported. For example, the “crude” Malthusian explanation, which connects popular immiseration to social breakdown, fails to account both for the start and end of the “Time of Troubles.” Many of the historical “Golden Ages” were the golden ages only for the elites, whose high levels of consumption were based on low real wages and falling consumption levels of the great majority of the population. Furthermore, while declining living standards are often a contributing factor to the social pressures for instability, reversing this trend does not end instability until elite overproduction is also reversed (more detailed discussion in Chapter 10 of Ref. [11]).

**Table 1:** Historical crises studied by structural-demographic theorists.

Crisis	Time (century)	Reference
English Civil War	XVII	[3]
French Revolution	XVIII	[3]
Ming-Qing Transition	XVII	[3]
Ottoman Crisis of XVII c.	XVII	[3]
European Revolutions of 1848–49	XIX	[3]
Tokugawa Crisis in Japan	XIX	[3]
Wars of the Roses in England	XV	[11]
Late Medieval Crisis in France	XIV–XV	[11]
Wars of Religion in France	XVI	[11]
Time of Troubles in Russia	XVII	[11]
Russian Revolution	XX	[11]
Crisis of the Late Republic in Rome	I BCE	[11]
Roman Principate Crisis	III	[11]
Roman Dominate Crisis	VI	[27]
American Civil War	XIX	[10]
Contemporary American Crisis	XXI	[10]
Chartist Crisis in Britain	XIX	[28]
Contemporary UK Crisis	XXI	[15]
Arab Spring in Egypt	XXI	[26, 29]
Overview of Crises in Medieval and Early Modern Egypt		[12]
Overview of Crises in Ancient Societies		[30]

These detailed investigations of a large (and growing) set of historical cases are valid scientific tests of the structural-demographic theory. Goldstone developed his theory by a careful quantitative analysis of the factors leading to the English Civil War of the seventeenth century and then tested the validity of his insight on several other examples of revolutions and rebellions in the early-modern world [for a historical retrospective, see 4]. While these initial tests had an element of circularity (because the data were used in theory construction), subsequent tests (the majority in Table 1) did not suffer from this potential problem, because they brought new data to bear on this question. Making scientific predictions about the events that happened, but are not known to the authors of the theory, is a valid scientific approach in historical sciences, such as geology, astrophysics, evolutionary biology, and cliodynamics (history as science). It is sometimes referred to as “retrodiction” [31].

The current article has expanded the set of empirical cases by adding to it an empirical test of prediction about the future. Such future-looking predictions cannot be the sole way to test theories of social macrodynamics (such as the SDT). Manipulative experiments in such disciplines are not possible for both ethical and practical reasons. For slowly developing processes (such as the structural-demographic ones) predictions must be about distant-enough future (ten years, as in our case, is really the minimal time). Finally, the modern world is thickly interconnected, and thus our cases studies are not truly independent ones. For these reasons, the primary way of testing theories in historical dynamics is retrodiction. But when mulitple successful tests using retrodiction (prediction about the past) are complemented with a few cases of prediction about the future, our degree of confidence in the theory is correspondingly enhanced.

The final comment that we wish to make is that the success of the 2010 SDT forecast offers some hope for our troubled times. The SDT is not merely a theory for understanding why internal violence outbreaks develop and spike. By providing us with the understanding of the deep structural causes of socio-political instability and societal breakdown, SDT also gives us tools for adopting the right set of reforms and policy interventions that can reverse these drivers of instability [1].

## Postscript

The analysis on which this article is based, and the first version that was submitted for review, were completed in January 2020. The year of 2020 has been a very eventful one, and in this Postscript we comment on how structural-demographic theory can make sense of these events. For conciseness, we focus on the USA.

The first point we need to make, however, is that all fundamental conclusions from our analysis are unchanged by what happened in 2020. Structural-demographic theory is concerned with social trends that develop slowly—on the scale of many years and decades. Thus, the 2010 forecast did not focus on any specific year, but rather on the whole decade. Our analysis has shown that various measures of instability increased during the period of 2011–18 (recollect that our data currently extends only into 2018). It appears likely that 2019 and, especially, 2020 will extend this rising trend. Furthermore, none of the fundamental structural-demographic drivers have been reversed, so far. Thus, the American social system continues to be very vulnerable to additional “quakes”.

Second, although the Covid-19 pandemic could not be, and was not predicted, it is important to note that disease outbreaks occur much more frequently during the crisis periods [32]. Such epidemics historically have had a disproportionate effect on the less advantaged segments of the population, and the Covid-19 pandemic was not an exception of this macrohistorical pattern. What this means in terms of structural-demographic theory is that the pandemic has further worsened the well-being of large swaths of the American population and, consequently, drove up the mass-mobilization potential. Furthermore, the governmental dysfunction in dealing with the pandemic, coupled with intra-elite infighting, will likely further depress the already low level of trust in government institutions. Thus, the effect of the shock delivered by the coronavirus has been to further destabilize the American polity.

Third, as we discussed in the Introduction, actual outbreaks of political violence require triggers, which in the case of May-June anti-government demonstrations and riots in the USA was the death of George Floyd at the hands of the Minneapolis police. Together with the rest of progressive humanity we hope that this death will not be in vain and will result in positive social change. The important point to keep in mind, however, is that until the fundamental SD drivers for instability are reversed, there will be other triggering events, which means that social turbulence may continue for years ahead. Historical data indicate that periods of enhanced instability and internal warfare usually extend for many years, with the median length in the 10–15 years range. On the other hand, as we noted at the end of discussion, the SDT gives us understanding of the deep structural causes of socio-political instability and, therefore, the tools for adopting the right set of reforms and policy interventions that can reverse these drivers for instability. It remains to be seen whether our society will be able to use these tools.

## Supporting information

S1 Data (CSV)

S1 File (ZIP)
